# Current prevalence and geographic distribution of helminth infections in the parasitic endemic areas of rural Northeastern Thailand

**DOI:** 10.1186/s12889-023-15378-4

**Published:** 2023-03-08

**Authors:** Pongsakorn Martviset, Wansika Phadungsil, Kesara Na-Bangchang, Wiwat Sungkhabut, Tanutchamon Panupornpong, Parisa Prathaphan, Nattaya Torungkitmangmi, Salisa Chaimon, Chompunoot Wangboon, Mantana Jamklang, Sirilak Chumkiew, Pichanee Watthanasiri, Amornrat Geadkaew-Krenc, Rudi Grams, Mathirut Mungthin, Pathanin Chantree

**Affiliations:** 1 Department of Preclinical Science, Faculty of Medicine, Thammasat University, Pathumthani, Thailand; 2 Thammasat University Research Unit in Nutraceuticals and Food Safety, Thammasat University, Pathumthani, Thailand; 3 Graduate Program in Biomedical Sciences, Faculty of Allied Health Sciences, Thammasat University, Pathumthani, Thailand; 4 Graduate Program in Bioclinical Sciences, Chulabhorn International College of Medicine, Thammasat University, Pathumthani, Thailand; 5 Office of Disease Prevention and Control Region-9, Ministry of Public Health of Thailand, Nakhon Ratchasima, Thailand; 6 Nakhon Ratchasima Provincial Health Office, Nakhon Ratchasima, Thailand; 7 Graduate Program in Biochemistry and Molecular Biology, Faculty of Medicine, Thammasat University, Pathumthani, Thailand; 8 Institute of Science, Suranaree University of Technology, Nakhon Ratchasima, Thailand; 9 Department of Parasitology, Phramongkutklao College of Medicine, Bangkok, Thailand

**Keywords:** Helminth infection, Rural areas, Geographic findings, *O. viverrini*

## Abstract

**Background:**

Helminth infection is a global health issue that not only causes acute helminthiasis but long-term infection may lead to complicated symptoms as well as severe complications. The World Health Organization cooperated with the Ministry of Public Health in many countries, particularly where high prevalence, spending a lot of resources for limiting the infection. In Thailand, the incidence of parasitic helminth infections was continuously declined in the last few decades according to several campaigns for parasitic elimination. However, the rural community in the northeast of Thailand where the highest prevalence of the country still needs to be monitored. This present study aims to report the current prevalence of parasitic helminth infections in Nakhon Ratchasima and Chaiyaphum provinces where sharing a huge area of the northeastern region of Thailand but only a few studies have been published.

**Methods:**

The stool specimens were collected from 11,196 volunteers and processed by modified Kato-Katz thick smear, PBS-ethyl acetate concentration techniques, and PCR. The epidemiological data were collected, analyzed, and used for generating of parasitic hotspots.

**Results:**

The results indicated that *O. viverrini* remains the major parasite in this area with a total prevalence of 5.05% followed by *Taenia* spp., Hookworms, *T. trichiura*, and *Echinostoma* spp., respectively. Mueang district of Chaiyaphum province has the highest prevalence especially *O. viverrini* with a prevalence of 7.15% that higher than the latest national surveillance. Interestingly, the prevalence of *O. viverrini* was hugely reported (more than 10%) in five subdistricts. The geographic localization of *O. viverrini* infections revealed that a lot of water reservoirs such as the lakes or branches of the river in the two-most prevalent subdistricts. Our finding indicated that gender and age were insignificantly different.

**Conclusion:**

This finding suggested that the parasitic helminth infection in the rural areas of northeast of Thailand remains high and the housing location is a major contributing factor for the parasitic infection.

## Background

Parasitic helminth infection remains a crucial global public health problem, especially in tropical and sub-tropical countries in several continents including Africa, South America, and Asia–Pacific [1]. In general, parasitic helminths have been classified into two major groups, roundworms (nematode) and flatworms (including trematodes and cestodes), which cause deleterious effects to humans [2]. For the roundworms, the most apprehension is soil-transmitted helminths (STHs) that comprised of three major parasites, large roundworm (*Ascaris lumbricoides*), hookworms (*Ancylostoma duodenale* and *Necator americanus*), and whipworm (*Trichuris trichiura*)*.* The global estimation of STHs infection was approximately 1.5 billion people in the last 10 years but nowadays the situation still endured [3, 4]. STHs can be transmitted effortlessly by contacting with soil, ingesting contaminated foods, vegetables, and fruits, also drinking water containing the infective stage of the parasite [5, 6]. The symptoms can be ranged from mild to severe complications such as mild anemia, malnutrition, chronic diarrhea, intestinal obstruction, parasitic dissemination, visceral organ failure, and occasional death. Other than STHs, *Strongyloides stercoralis* and lymphatic filarial parasites are also important nematodes that can cause serious effects to humans by causing dissemination and lymphatic elephantitis, respectively [1].

For the flatworm parasites, the trematodes or flukes remain the major infective organisms with the estimation of more than 300 million infected people globally [3]. *Schistosoma* spp., *Opisthorchis viverrini*, and *Clonorchis sinensis* are major parasites in this category. *Schistosoma* spp. are blood flukes that are transmitted to humans by contacting natural water containing infective cercariae while *O. viverrini*, and *C. sinensis* are foodborne parasites transmitted to humans by ingesting metacercariae (an infective stage) from undercooked fish especially Cyprinoid fish [2, 3, 7]. The severe complication of *O. viverrini*, and *C. sinensis* infections is cholangiocarcinoma (CCA), a bile duct cancer, that is highly aggressive cancer with a poor prognosis and leads to death in a short period [8, 9]. These parasites are endemic in different parts of the world, *O. viverrini* is distributed mainly in Southeast Asian countries, especially Laos PDR, Cambodia, Vietnam, also Thailand while *C. sinensis* is the major parasite in China, Korea, and Japan [10]. Other important flatworm parasites are cestodes or tapeworms that comprised of several species such as *Taenia* spp., *Hymenolepis* spp., *Echinococcus* spp., etc. The estimation of Tapeworm infection is greater than 50 million infected peoples worldwide, especially in poor sanitation communities that incidentally cause serious effects such as neurocysticercosis, seizure, and death [11].

Thailand is a tropical country located in the Great Mekong Subregion (GMS) of Southeast Asia where a huge of parasitic infection cases have been continuously reported. The most important so far was *O. viverrini* that endemic mainly in the northeastern and northern areas. The highest prevalence was firstly reported in 1955 with 100% prevalence in certain villages of Khon Kaen province [12], and 30-year later (1985) the prevalence remained 100% in some villages of Chonnabot district, Khon Kaen province [13]. The first nationwide survey has been reported during 1980–1981 that the overall prevalence of *O. viverrini* was 14% with the highest percentage in the northeast of 34.6% [14]. The prevalence was continuously reduced over the year due to several campaigns of liver fluke elimination of the Ministry of Public Health (MOPH) of Thailand. Consequently, the nationwide prevalence was down to 9.4% in 2002 [15], 8.7% in 2009, 5.1% in 2014 [16], and recently 2.2% in 2019 [17]. The prevalence in the northeastern region was also decreased from 34.6% in 1980–1981 to 4.98% in 2019. However, some studies suggested that the prevalence was remaining high in some villages up to more than 20% not only in northeastern but also in northern and central areas of Thailand [18–20]. Apart from *O. viverrini*, STHs is another concern in Thailand. The national survey in 2009 revealed the prevalence of STHs of 7.7% including 6.5% of hookworm, 0.5% of *Ascaris lumbricoides*, and 0.7% of *Trichuris trichiura*. The highest prevalence was reported from the south (21.4%) followed by northeast of 5.1%, north 4.7%, and central 3.8%, respectively [16]. However, the prevalence in some areas was ranging from 0—18.4% depending on age, occupation, behavior, and sanitation [20–24].

**Fig. 1:**
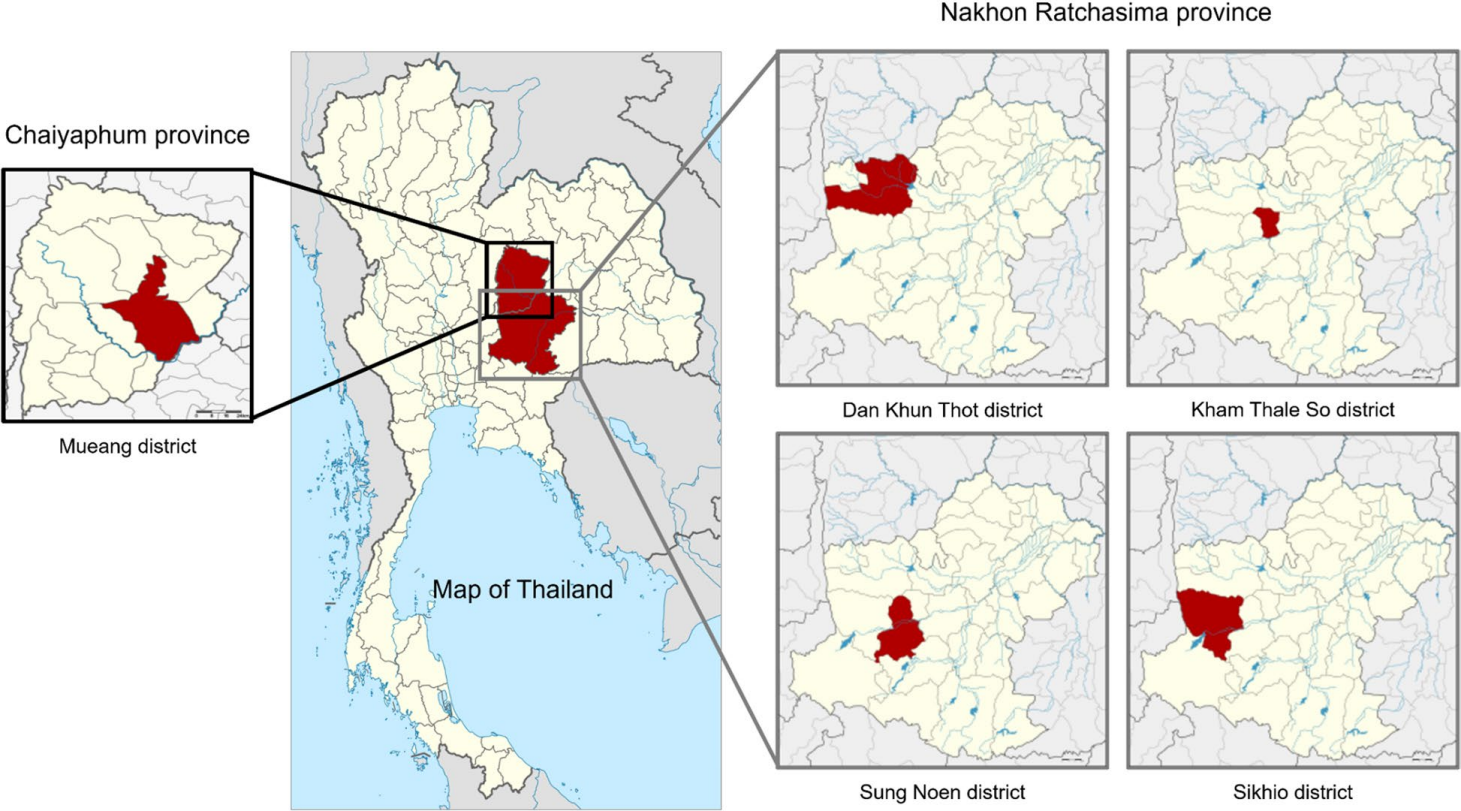
Map of five districts where are the study sites of this study located in Nakhon Ratchasima and Chaiyaphum provinces, Northeastern region of Thailand. (Map from https://commons.m.wikimedia.org/wiki/Atlas_of_Thailand)

Nakhon Ratchasima and Chaiyaphum provinces are the provinces that locating in the parasitic endemic area of the northeast of Thailand (Fig. 1.) where housing of more than 3.4 million people and shared approximately 14% of the northeastern population (National Housing Authority of Thailand, 2020). Even it is a very huge area but only a few studies have been reported and most of them were focused on the *O. viverrini* infection, concentratedly, Nakhon Ratchasima province [25–27]. The present study would like to investigate the current parasitic infection status of the population in these areas covering 4 districts of Nakhon Ratchasima province and one big district of Chaiyaphum province that has never been reported. Moreover, the geographic localization will be applied to pointed in the subdistricts where high prevalence was found.

## Materials and methods

### Study areas

The study sites were located in 4 districts of Nakhon-Ratchasima province (including Dan Khun Thot, Kham Thale So, Sung Noen, and Sikhio districts) and one district of Chaiyaphum province (Mueang district), northeast of Thailand (Fig. 1). All five districts are neighboring connected areas that cover more than 4,830 km^2^ including 60 subdistricts with more than 580 villages, where the housing of more than 542-thousands people in 2020 (National Housing Authority of Thailand, 2020). These areas are located in the border between northeastern and central regions of Thailand where most rice farming areas in the rainy season for approximately five months but extremely dry in the rest of the year. Mueang district of Chaiyaphum province has a little different geography due to a lot of canals branching from Chi river, one of the longest rivers in the northeast of Thailand.

### Specimen collection, modified Kato-Katz thick smear, and PBS-ethyl acetate concentration technique (PECT)

The stool samples were collected from 11,196 volunteers who live in that areas of all ages, sexes, and occupations from January to June 2021. In brief, the volunteers were educated for non-invasive stool collection and the stool specimens were collected for approximately 5–10 g in the leak-proofed containers then immediately sent to the laboratory or kept in 4–10 °C until the process. The fresh specimens were investigated for parasitic infection by modified Kato-Katz thick smear [28]. In brief, the stool specimens were filtered through a 0.5–1 mm grille for removing debris and dietary fibers. The filtered stool was then transferred to the glass slide for approximately 100–150 mg and imbricated with cellophane soaked with glycerin-malachite green and pressed to equalize the specimen then allowed to dry for 15–30 min before being observed under a light microscope.

The positive samples from modified Kato-Katz thick smear were subjected to sedimented by PBS-ethyl acetate concentration technique (PECT) as previously described [29–31] with a few modifications. In brief, 0.5–1 g of stool specimen was mixed with 10 mL of 0.01 M PBS, pH 7.4, and filtered through three layers of sterile gauze pad. Three mL of ethyl acetate was added to the filtrate and mixed thoroughly by inverting. The tube was then centrifuged at 2,000 × *g*, for 10 min and the two upper phases were discarded. The sediment was investigated under a light microscope.

### Genomic DNA isolation

The samples containing *O. viverrini* (*Ov*)-like eggs from both modified Kato-Katz thick smear and PECT were performed genomic DNA isolation by using PECT sediments as previously described. Before extraction, the sediments were brought to autoclave at 121 °C, for 5 min to open the operculum of operculated parasite eggs. The genomic DNAs were then extracted by using QIAamp® PowerFecal® DNA kit (QIAGEN, Germany) according to the manufacturer’s instructions with the final filtrates containing 50–100 µL of eluted DNAs.

### PCR amplification of OvNad5

The morphological characteristics of *O. viverrini* (*Ov*) and minute intestinal flukes (MIFs) eggs are similar. So, the *Ov* egg was confirmed by specific PCR amplification from genomic DNA using *Ov*Nad5 primer as previously described [30]. PCR amplifications were done by using GoTaq® Colorless Master Mix (Promega, USA) containing 3 µL of genomic DNA, and 25 pmol of each forward and reverse primer (*OvNad*5*-*F: TTTGCGGAGGTTTGTTACCT and *OvNad*5*-*R: CACCTCACCAATTCAACACG) in a thermal cycler (Mastercycler nexus Eppendorf flexlid, Germany). The amplification steps were including initial denaturation at 95ºC for 5 min, followed by 35 cycles of denaturation at 95ºC for 1 min, annealing at 55 ºC for 1 min, extension at 72 ºC for 1 min, and one cycle of a final extension at 72 ºC for 10 min. The PCR products were size separated on 2% agarose gel containing ViSafe Red Gel Stain (Vivantis, USA) using 1X TBE buffer at 80 V for 2 h. The PCR products were confirmed for their correction by DNA sequencing service (Macrogen, Republic of Korea).

### Geographic localization of parasitic hotspot

The geographic investigation of parasitic helminths was done in the subdistricts with a prevalence for any parasite of more than 20%. The village headman located the parasite-positive volunteer’s houses and the GIS data including longitude and latitude of the houses were collected by using Handheld GPS (Garmin eTrex 32x, Lenexa, KS, USA) with experimental uncertainty of ± 5 m. The obtained house locations of parasite-positive volunteers were imported to ArcGIS desktop software. The hotspots were analyzed using ArcMap version 10.3 and laid to the Google Earth map to illustrate the hotspots, including the geographic characteristics of villages, water reservoirs, rice fields, highlands, and mountains. The cluster analysis was performed to evaluate the relationship between the geographic findings and parasite-positive volunteer house locations by spatial autocorrelation using Global Moran’s I. *p-value* less than 0.05 was considered statistically significant.

### Statistical analysis

The data were entered into Microsoft Excel and double-checked to validate all data before analytical processing. All data analyses were performed using SPSS for Windows, Version 28.0 (SPSS, Chicago, IL). The prevalence of the parasites was described using percentages. For the age of volunteers, the participating individuals were classified into four age groups: 0–20, 21–40, 41–60, and older than 60 years. Univariate and multivariate logistic regression analyses were performed to determine the associated factors, including sex and age interval. The magnitudes of the associations obtained from univariate and multivariate analyses are represented as crude odds ratios (CORs) and adjusted odds ratios (AORs), respectively, with the corresponding 95% CIs. Fisher's exact test was used to assess differences in the presence of parasites by age group. *p-value* less than 0.05 was considered statistically significant.

## Results

### Prevalence of parasitic helminth infections

From 11,196 stool samples, five parasitic helminths were found with varied prevalence. The highest overall prevalence was *O. viverrini* (OV) single infection with the prevalence of 5.02% (562/11,196) (total prevalence of *O. viverrini* was 5.05% [565/11,196] when OV + TN [*Taenia* spp.] positive samples were included), followed by hookworm (HW) with a prevalence of 0.20% (22/11,196), *Taenia* spp. (TN) single infection was 0.42% (47/11,196) (0.45% [50/11,196] when OV + TN positive samples were included), *T. trichiura* (TT) infection was 0.10% (11/11,196), *Echinostoma* spp. (ES) infection was 0.01% (1/11,196), and OV + TN mixed infection prevalence was 0.03% (3/11,196). The rest of the population was reported as negative for all parasites with the proportion of 94.23% (10,550/11,196).

In Nakhon-Ratchasima province, the prevalence of OV infection was highest in Dan Khun Thot district (1.68%, 23/1,368) followed by Sikhio (1.09%, 11/1,012), Sung Noen (0.38%, 4/1,065), and Kham Thale So (0.24%, 1/409), respectively. Interestingly, one subdistrict (Ban Kao subdistrict) of Dan Khun Thot district had the highest prevalence of OV infection of 6.32% (6/95). For the other parasites, the prevalence in all districts was lower than 1%. The prevalence of HW infection was highest in Sung Noen (0.66%, 7/1,065), followed by Sikhio (0.49%, 5/1,012), Dan Khun Thot (0.29%, 4/1,368), and Kham Thale So (0.24%, 1/409) districts, respectively. The prevalence of TN infection was highest in Kham Thale So (0.98%, 4/409), followed by Sikhio (0.49%, 5/1,012), Dan Khun Thot (0.22%, 3/1,368), and Sung Noen (0.09%, 1/1,065). For TT infection, Sikhio district was the highest prevalence of 0.20% (2/1,012) followed by Dan Khun Thot (0.15%, 2/1,368), and Sung Noen (0.09%, 1/1,065), respectively. Contrary, TT was not found in Kham Thale So district. Moreover, ES was found only in Sikhio district with a prevalence of 0.10% (1/1,012).

**Fig. 2:**
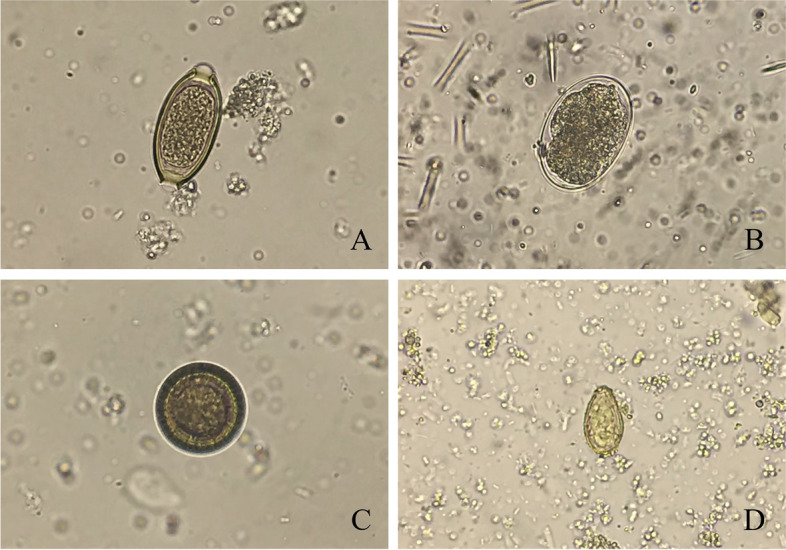
The parasite eggs found in the PECT sediments at 400X magnification. **A**; *Trichuris trichiura* (TT) egg, **B**; hookworm (HW) egg, **C**; *Taenia* spp. (TN) egg, **D**; *Opisthorchis viverrini* (OV) egg

In Mueang district of Chaiyaphum province, the highest occurring parasitic infection was OV infection with a prevalence of 7.15% (526/7,344) followed by TN (0.49%, 7/7,344), TT (0.08%, 6/7,344), and HW (0.07%, 5/1,344). OV infection in this district was interestingly considerable, especially when focused on the subdistricts with a prevalence of more than 10%. The top 5 highest prevalence subdistricts were Huai Ton (25.88%, 117/452), Lat Yai (22.38%, 94/420), Na Siao (12.86%, 53/412), Na Fai (12.84%, 57/444), and Phon Thong (12.27%, 53/432) subdistricts, respectively. The prevalence data is shown in Table 1 and the parasite eggs found in the PECT sediment are illustrated in Fig. 2.

**Table 1:** Prevalence of parasitic infection detected in the stool specimens from Nakhon Ratchasima and Chaiyaphum provinces by Kato-Katz thick smear and PECT. (OV, *O. viverrini*; HW, Hookworm; TN, *Taenia* spp.; TT, *T. trichiura*; ES, *Echinostoma* spp.)

**District/Province**	**Subdistrict**	**No. of collected samples**	**No. of positive samples**						**No. of negative samples**	**Prevalence (%)**
			**OV**	**HW**	**TN**	**TT**	**ES**	**OV+TN**		
Dan Khun Thot/ Nakhon Ratchasima	Ban Praeng	98	2	0	0	0	0	0	96	OV = 1.68 HW = 0.29 TN = 0.22 TT = 0.15 ES = 0.00
	Nong Krat	75	1	0	0	0	0	0	74	
	Dan Nok	100	3	0	1	0	0	0	96	
	Nong Sai	95	1	2	0	0	0	0	92	
	Nong Bua Lakhon	67	2	1	0	0	0	0	64	
	Hin Dat	95	1	0	1	0	0	0	93	
	Dan Nai	80	3	0	1	0	0	0	76	
	Kut Phiman	95	3	0	0	1	0	0	91	
	Ban Kao	95	6	0	0	0	0	0	89	
	Dan Khun Thot	92	1	1	0	0	0	0	90	
	Takhian	87	0	0	0	0	0	0	87	
	Sa Chorakhe	100	0	0	0	0	0	0	100	
	Nong Bua Takiat	100	0	0	0	0	0	0	100	
	Huai Bong	97	0	0	0	0	0	0	97	
	Non Muaeng Patthana	92	0	0	0	1	0	0	91	
	**Total**	**1368**	**23**	**4**	**3**	**2**	**0**	**0**	**1336**	
Kham Thale So/ Nakhon Ratchasima	Pong Daeng	107	0	0	2	0	0	0	105	OV = 0.24 HW = 0.24 TN = 0.98 TT = 0.00 ES = 0.00
	Bueng O	111	1	1	0	0	0	0	110	
	Kham Thale So	102	0	0	1	0	0	0	101	
	Phun Dung	88	0	0	1	0	0	0	87	
	**Total**	**409**	**1**	**1**	**4**	**0**	**0**	**0**	**403**	
Sung Noen/ Nakhon Ratchasima	Sung Noen	77	1	1	0	0	0	0	75	OV = 0.38 HW = 0.66 TN = 0.09 TT = 0.09 ES = 0.00
	Sema	103	0	1	0	1	0	0	101	
	Khorat	107	0	0	0	0	0	0	107	
	Bung Khilek	103	1	0	0	0	0	0	102	
	Non Kha	107	0	0	0	0	0	0	107	
	Khong Yang	99	0	0	0	0	0	0	99	
	Makluea Kao	95	1	1	1	0	0	0	92	
	Makluea Mai	87	0	2	0	0	0	0	85	
	Na Klang	101	1	0	0	0	0	0	100	
	Nong Takai	99	0	0	0	0	0	0	99	
	Kud Chik	87	0	2	0	0	0	0	85	
	**Total**	**1065**	**4**	**7**	**1**	**1**	**0**	**0**	**1052**	
Sikhio/ Nakhon Ratchasima	Sikhio	66	1	0	0	0	0	0	65	OV = 1.09 HW = 0.49 TN = 0.49 TT = 0.20 ES = 0.10
	Ban Han	58	2	0	0	0	0	0	56	
	Kritsana	91	1	0	0	0	0	0	90	
	Lat Bua Khao	116	0	0	1	1	0	1	113	
	Nong Ya Khao	90	0	1	0	1	0	0	88	
	Kut Noi	94	1	1	1	0	0	0	91	
	Nong Nam Sai	78	0	0	0	0	1	0	77	
	Wang Rong Yai	98	1	1	0	0	0	0	96	
	Mittraphap	122	2	2	2	0	0	0	116	
	Don Mueang	100	0	0	0	0	0	0	100	
	Nong Bua Noi	99	2	0	0	0	0	0	97	
	**Total**	**1012**	**10**	**5**	**4**	**2**	**1**	**1**	**989**	
Mueang/ Chaiyaphum	Phon Thong^a^	432	53	0	1	0	0	1	377	OV = 7.15 HW = 0.07 TN = 0.49 TT = 0.08 ES = 0.00
	Huai Ton^a^	452	117	0	0	0	0	0	335	
	Na Fai^a^	444	57	0	3	0	0	0	384	
	Lat Yai^a^	420	94	3	5	0	0	0	318	
	Na Siao^a^	412	53	1	9	3	0	1	344	
	Ban Khai	420	17	0	0	1	0	0	402	
	Kut tum	415	10	0	0	1	0	0	404	
	Chi Long	372	15	1	2	0	0	0	354	
	Ban Lao	380	14	0	1	1	0	0	364	
	Nong Na Saeng	404	15	0	2	0	0	0	387	
	Nong Phai	400	11	0	4	0	0	0	385	
	Tha Hin Ngom	401	13	0	2	0	0	0	386	
	Huai Bong	302	12	0	1	0	0	0	289	
	Non Samran	320	5	0	1	0	0	0	314	
	Khok Sung	365	10	0	2	0	0	0	353	
	Bung Khla	412	12	0	0	0	0	0	400	
	Sap Si Thong	389	11	0	2	0	0	0	375	
	Nai Mueang	284	2	0	0	0	0	0	282	
	Rop Mueang	320	3	0	0	0	0	0	317	
	**Total**	**7344**	**524**	**5**	**35**	**6**	**0**	**2**	**6770**	
**Overall (n) (%)**		**11196**	**562 (5.02)**	**22 (0.20)**	**47 (0.42)**	**11 (0.10)**	**1 (0.01)**	**3 (0.03)**	**10550 (94.23)**	

^a^Top 5 subdistricts with the highest prevalence of *O. viverrini* infection: Huai Ton; 25.88%, Lat Yai; 22.38%, Na Siao; 12.86%, Na Fai; 12.84%, and Phon Thong; 12.27%, respectively

### Epidemiological data

In this study, the basic information of the volunteers was collected including sexes and ages. From 11,196 participants, the female was the major population of 66.75% (7473/11196) and the male was 33.25% (3723/11196). In the parasite positive group, the female was the major proportion of 63.31% (409/646) with the male population of 36.69% (409/646). The chi-square test revealed that the sexes in this group were statistically significant (*p* < *0.05*, data not shown) but when compared to the parasite negative group, the proportion was similarly equal with 66.96% (7064/10550) female and 33.04% (3486/10550) that made the insignificant difference of both crude odds ratio (COR) (*p* = *0.553*) and adjusted odds ratio (AOR) (*p* = *0.402*). Interestingly, the relationship between gender and parasitic infection was statistically significant in Kham Thale So and Sung Noen districts (COR *p-value* = *0.024* and *0.039*, respectively). Females in these two districts had low risk than the other subdistricts with a relative risk (RR) of only 1.09% and 0.95%, respectively, while in the other districts the RRs were more than 2% and reached up to 5.47% in overall female participants. Moreover, the RRs of male and female participants (6.37% and 5.47%, respectively) in our study were statistically insignificant (*p* > *0.100*) as shown in Table 2.

**Table 2:** General characteristics of participants from 5 districts divided by status of parasitic infection

**District**		**Dan Khun Thot**		**Kham Thale So**		**Sung Noen**		**Sikhio**		**Mueang Chaiyaphum**		**Overall**		**Relative risk by gender**
**Parasitic infection status (positive with at least one parasite)**		**Positive n (%)**	**Negative n (%)**	**Positive n (%)**	**Negative n (%)**	**Positive n (%)**	**Negative n (%)**	**Positive n (%)**	**Negative n (%)**	**Positive n (%)**	**Negative n (%)**	**Positive n (%)**	**Negative n (%)**	
Sex	Male	14 (43.75)	518 (38.77)	3 (50.00)	130 (32.26)	6 (46.15)	322 (30.61)	7 (30.43)	281 (28.41)	207 (36.19)	2235 (33.01)	237 (36.69)	3486 (33.04)	Male: 6.36
	Female	18 (56.25)	818 (61.23)	3 (50.00)	273 (67.74)	7 (53.85)	730 (69.39)	16 (69.57)	708 (71.59)	365 (63.81)	4535 (66.99)	409 (63.31)	7064 (66.96)	Female: 5.47
	COR (95% CI) *p-value*	1.229(0.700-2.159) 0.473		2.125 (1.196-3.775) 0.010*		1.896 (1.064-3.380) 0.030*		1.102 (0.598-2.031) 0.755		1.142 (0.637-2.047) 0.655		1.192 (0.666-2.134) 0.553		
	AOR (95% CI) *p-value*	1.211 (0.808-1.815) 0.354		1.598 (1.064-2.400) 0.024*		1.541 (1.023-2.320) 0.039*		1.149 (0.754-1.750) 0.519		1.167 (0.773-1.762) 0.462		1.190 (0.790-1.799) 0.4002		
Age interval	0-20	0 (0.00)	4 (0.30)	0 (0.00)	0 (0.00)	0 (0.00)	9 (0.86)	1 (4.35)	79 (7.99)	33 (5.77)	413 (6.10)	34 (5.26)	505 (4.79)	6.31
	21-40	3 (9.38)	138 (10.33)	1 (16.67)	25 (6.20)	0 (0.00)	97 (9.22)	6 (26.09)	113 (11.43)	99 (17.31)	609 (9.13)	109 (16.87)	982 (9.31)	10.89
	41-60	20 (62.50)	914 (68.41)	4 (66.67)	257 (63.77)	10 (76.92)	606 (57.60)	11 (47.83)	589 (59.56)	370 (64.69)	4062 (60.00)	415 (64.24)	6428 (60.93)	6.06
	>60	9 (28.13)	280 (20.96)	1 (16.67)	121 (30.02)	3 (23.08)	340 (32.32)	5 (21.74)	208 (21.03)	70 (12.24)	1686 (24.90)	88 (13.62)	2635 (24.98)	3.23
	Total	32	1336	6	403	13	1052	23	989	572	6770	646	10550	
	*Fisher’s Exact Test value*	1.182		9.128		13.987		8.931		7.129		5.442		
	*p-value*	0.556		<0.001*		<0.001*		0.014*		0.059		0.130		

**Fig. 3:**
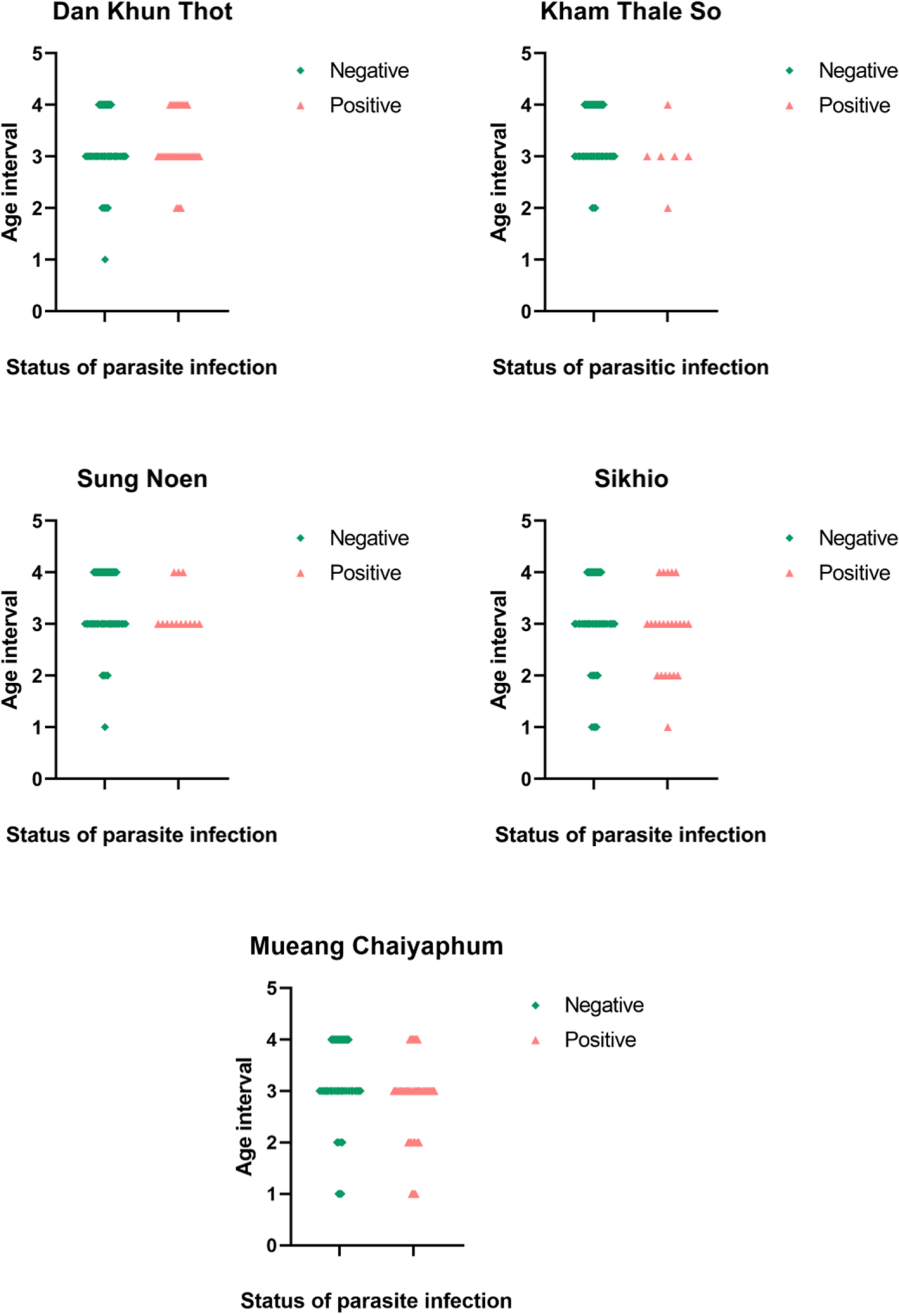
Distribution of the age intervals between parasite-positive and -negative participants in all five districts. The result indicated insignificant differences among the districts used in our study

The age of participants was separated in four groups (1; 0–20, 2; 21–40, 3; 41–60, 4; > 60 years). From overall collected data, in the parasite positive group, the highest prevalence was reported from age 41–60-year-old with 64.24% (415/646) followed by 21–40-year-old with 16.87% (109/646), > 60-year-old with 13.62% (88/646), and 0–20-year-old with 5.26% (24/646). However, the proportion was insignificantly different (*p* = *0.130*) from parasite negative group which comprised of 60.93% (6428/10550) of 41–60-year-old followed by 9.31% (982/10550) of 21–40-year-old, 24.98% (2635/10550) of > 60-year-old, and 4.79% (505/10550) of 0–20-year-old, respectively. When focusing on the proportion in each district, the trend in all districts was similar as illustrated in Fig. 3., however, there are statistically significant differences in Kham Thale So, Sung Noen, and Sikhio districts but the major populations were still from the age of 41–60-year-old. The relative risk was highest in the age between 21–40-year-old (10.89), followed by 0–20-year-old (6.31), 41–60-year-old (6.06), and > 60-year-old (3.23), respectively. The epidemiological data is shown in Table 2.

### Geographic distribution of O. viverrini (OV), the major parasite from the present study

**Fig. 4:**
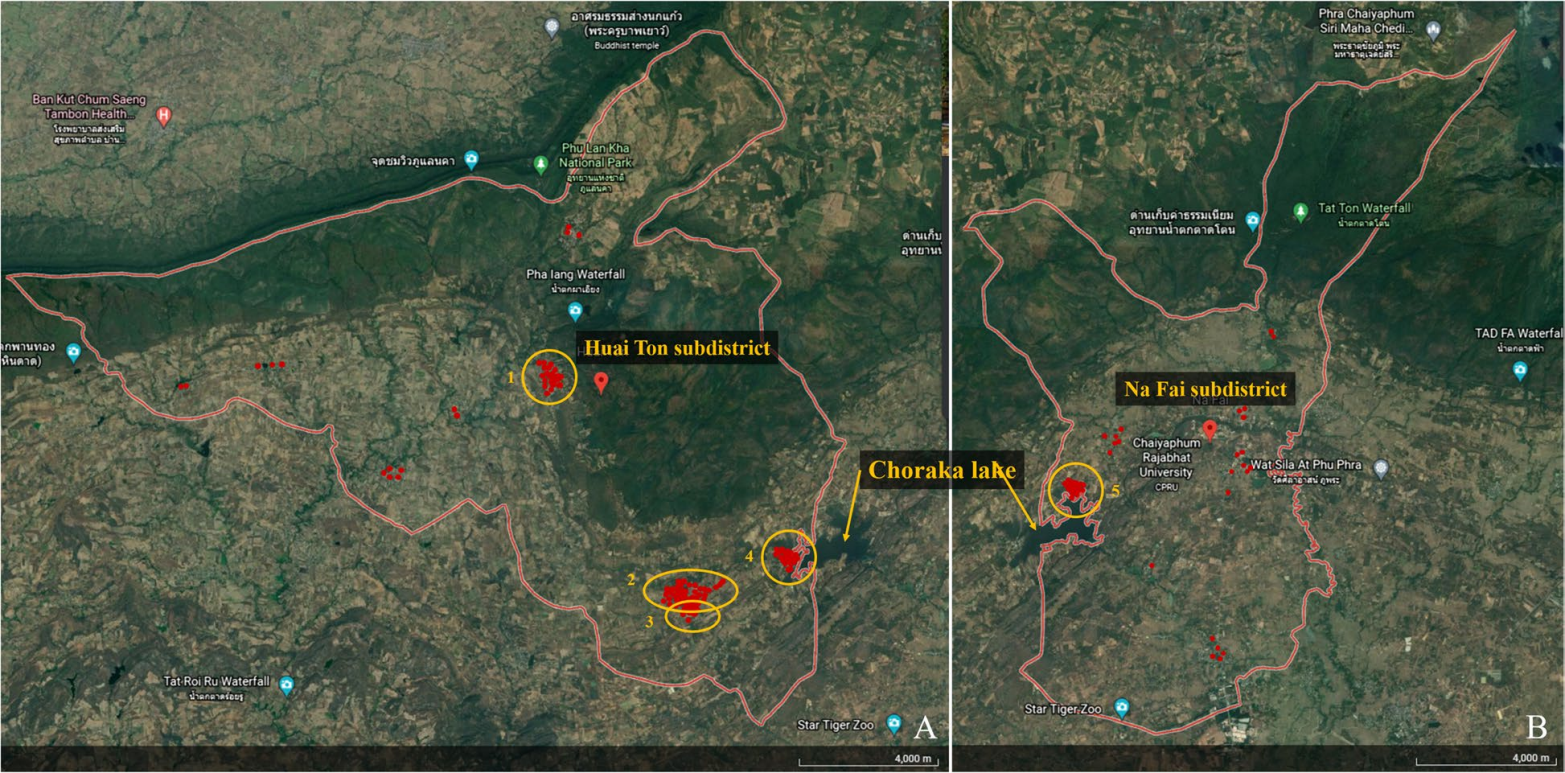
Geographic distribution of OV positive participants in the two connected subdistricts of Mueang district, Chaiyaphum province. **A**; Huai Ton subdistrict, **B**; Na Fai subdistrict. The red spots located the OV positive participant’s houses and the yellow circles remark the village with high prevalence (1; Chi Long Nuea, 2; Huai Ton, 3; Pong Khong Tai, 4; Pong Khong Nuea, 5; Nong Nok Khao)

The highest prevalence was reported from Huai Ton (25.88%), followed by Lat Yai (22.38%), Na Siao (12.86%), Na Fai (12.84%), and Phon Thong (12.27%) subdistricts, respectively. The collected household data were then used for localizing the hotspot of OV infection in these subdistricts. Our findings suggested that most of the infected persons in Huai Ton subdistrict habitat in four villages (Huai Ton, Pong Khong Nuea, Pong Khong Tai, and Chi Long Nuea) as illustrated in Fig. 4. Three out of four villages, including Huai Ton, Pong Khong Nuea, and Pong Khong Tai were closely related to one reservoir called “Choraka lake”. Pong Khong Nuea village was the only village located next to the lake, while Huai Ton and Pong Khong Tai were located 3–4 km away from the lake but these villages were connected to the lake by the canal. Moreover, in Na Fai subdistrict, the fourth-highest prevalence, where located next to Huai Ton subdistrict also connected with Choraka lake was found the hotspot of OV in one village called Nong Nok Khao where located in Choraka lake circumference. In Chi Long Nuea village, another village in Huai Ton subdistrict, where located in the north was different from the other villages. This village contains several small ponds and a canal that originated from Pha Iang waterfall but is not connected to the lake as shown in Fig. 4.

**Fig. 5:**
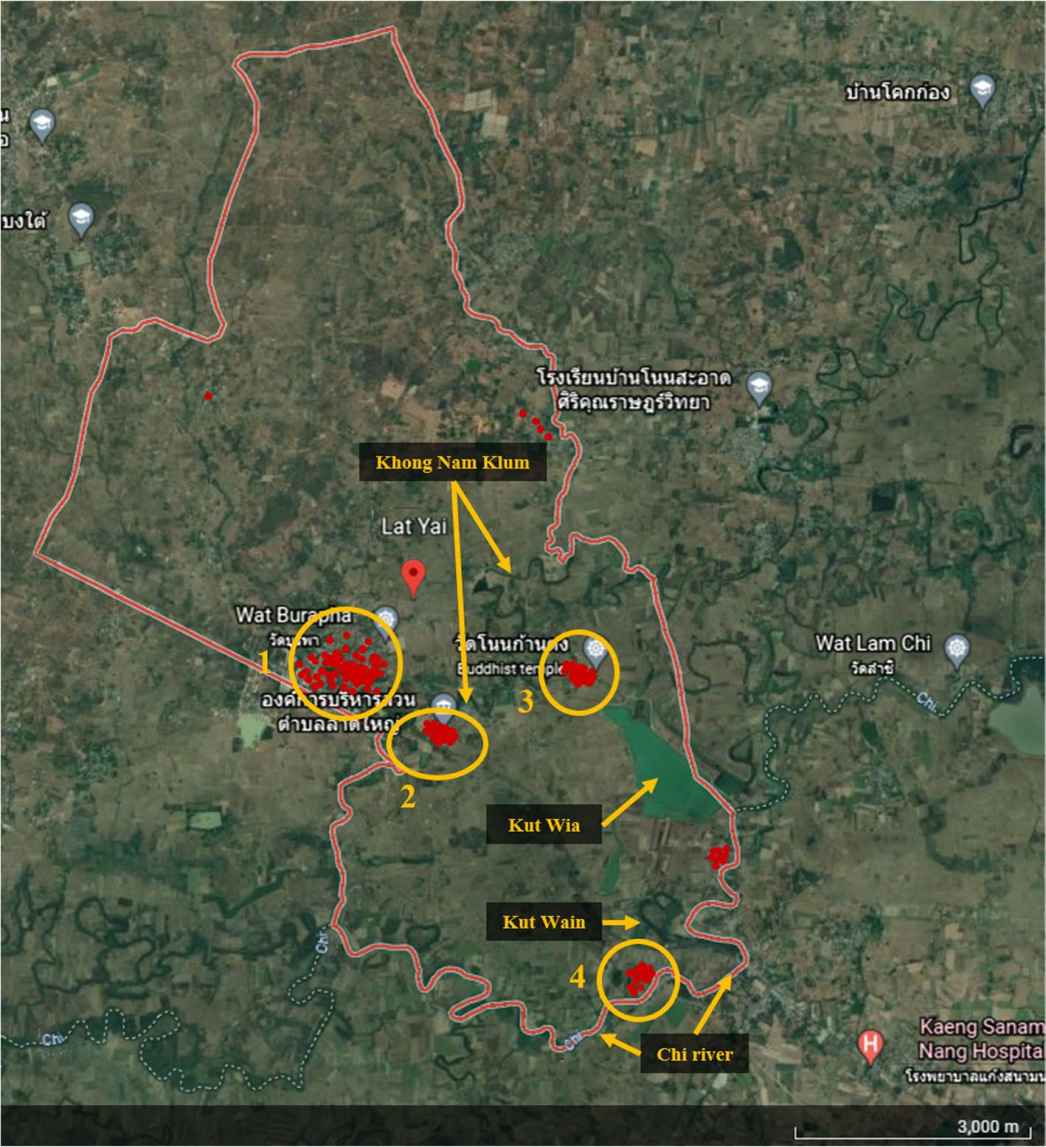
Geographic distribution of OV positive samples in Lat Yai subdistrict of Mueang district, Chaiyaphum province, the second-highest prevalence of OV infection. The red spots located the OV positive participant’s houses and the yellow circles remark the village with high prevalence (1; Lat Yai, 2; Non Wan Plai, 3; Non Kan Trong, 4; Kut Wian)

The second highest prevalence of OV was occurring in Lat Yai subdistrict. In this subdistrict, the hotspots were concentrated in the villages that are closely located next to the main and the branches of Chi river, including Lat Yai, Kut Wian, Non Wan Plai, and Non Kan Trong villages (*p* < *0.05* in all villages). In this subdistrict, the Chi river generated a lot of small reservoirs and canals, such as Khong Nam Klam, Kut Wai, Kud Pla Mun, Kut Wian, etc., as illustrated in Fig. 5.

## Discussion

Parasitic helminth infection is one of the major global health issues that is not only the cause of death but also the genesis of economic losses worldwide from the past to present [32, 33]. WHO has estimated that more than 2 billion people are infected with parasitic organisms, majority of helminths including soil-transmitted helminths (STHs) and flatworm parasites, especially *Schistosoma* spp. and carcinogenic liver flukes [3]. Thailand is the country located in the Great Mekong Subregion (GMS) of Southeast Asia that recognized as one of the highest prevalence of parasitic helminths infection over the last few decades with the considerable parasites including *O. viverrini* in north and northeast and STHs in the south [16]. The roadmap for limiting the parasitic infection of the Ministry of Public Health of Thailand has been proposed in 2014 including the campaign for elimination of the major parasitic organisms by 2025, especially *O. viverrini* and malaria (National health Assembly, Ministry of Public Health of Thailand, 2014). However, the parasitic infection status in the endemic areas still needs to be monitored. Our present study reports the currentstatus of parasitic infection in the northeast of Thailand where is an endemic area of several parasites. The study sites were included Nakhon Ratchasima and Chaiyaphum provinces which are the 1^st^ and 3^rd^ biggest provinces in terms of area size of the northeast of Thailand, respectively, where is a huge communitarian approximately 14% of the northeastern population. Unexpectedly, only a few reports indicate the parasitic situation in these areas, particularly, Chaiyaphum province.

We collected a large number of specimens (11,196) from those provinces. The findings indicated that *O. viverrini* remains the major parasite in this area but the other intestinal parasites were almost disappeared. The prevalence of *O. viverrini* in our study was 5.05% (including mixed infection) which is higher than the national surveillance reported in 2019 of 2.2% and also a little higher than the overall prevalence in the northeastern area (4.98%) [17]. The geographic information system (GIS) used in our study provided important information on parasitic epidemiology; rousingly, the parasitic hotspots appeared when the data were analyzed. Moreover, it illustrated the characteristics of the areas with a high prevalence of parasitic infections comprising of different geography from the other areas, especially the water reserves.

When focusing on Nakhon Ratchasima province, the *O. viverrini* prevalence of our study was only 1.01% (with the highest prevalence in Dan Khun Thot district of 1.68%) that lower than the previous studies. Kaewpitoon et al. reported in 2012 that the total prevalence of *O. viverrini* in Nakhon Ratchasima province was 2.48% and the highest prevalence was occurring in Non Daeng district of 16.67% followed by Pra Thai (11.11%), Lam Ta Men Chai (8.33%) and Kaeng Sanam Nang (8.33%), respectively. In the same area of our study, they reported that the prevalence in Dan Khun Thot district was 2.78%. In contrast, they did not find any positive samples in Sikhio, Sung Noen, and Kham Thale So districts [25]. However, the explanation due to that previous study is the sample size that was very small with only 36–40 samples of each district. The lowering prevalence found in our study will be related to several factors, especially the elimination campaign from the Ministry of Public Health of Thailand by administering of anti-helminthic drug as prophylactic agent annually. Moreover, the geography of this region was very dried when comparing to the other districts used in the previous study, such as Non Daeng and Pra Thai districts where located nearby the Mun river. In our study sites, four districts of Nakhon Ratchasima, there are only a few water reservoirs without main rivers, especially in Sung Noen and Kham Thale So districts. It is probable factors contribute to the different prevalence in this area.

The prevalence of *O. viverrini* in Chaiyaphum province in our study was different from Nakhon Ratchasima province. We found that the *O. viverrini* prevalence was higher reached to 7.15%, interestingly, five subdistricts were a prevalence of more than 10%. The present study is the first report from this area of Chaiyaphum province, surprisingly, it has a higher prevalence than expectation also when compared with the latest national surveillance [17]. Concentratedly, the top five highest prevalence subdistricts are located in the area which nearby the lakes, canals, river, and rivulets. It corresponds to previous reports that the humidity and water reservoirs are the main contributors to the parasite life cycle [34–38]. Even the occupations in our study were not different among the parasite positive and negative populations, but normally, the people in this area are doing other activities such as fishing for the rest of the year after finishing the rice farming season might be increased the risk of infection as well. Interestingly, in the highest prevalence subdistrict, Huai Ton, the houses of infected peoples were located near the Choraka lake where is the main water reservoir in Huai Ton and Na Fai subdistricts. This lake will be served as the natural source of the parasite and made the completion of the parasitic life cycle due to it being closely located to the villages where the parasite eggs can be passed from humans or other reservoir hosts [39].

Moreover, our finding also suggested that the Chi river located in Lat Yai subdistrict where the second highest prevalence (22.38%) is the same river upstream to Chonnabot district, Khon Kaen province where a lot of reports of a high prevalence of *O. viverrini,* especially in the villages nearby Lawa lake [13, 40–44]. Chi river is the longest river of the northeast of Thailand and also one of the main rivers of this region that originated from the mountain in Chaiyaphum province and flows passing to Khon Kaen, Mahasarakham, Roi-Et, Yasothon, and Ubon Ratchathani provinces then finally incorporated with Mekong river in the Thai-Laos border. Along the river, it generates a lot of rivulets, canals, and lakes that proper for the reproduction of fish and aquatic organisms. So, it is quite confirmation that the habitats where closely related to this river might be the factor that increases the risk of infection in this region and we do suggest that the water reservoirs generated from the Chi river will be huge source of infective parasite. Interestingly, no minute intestinal flukes (MIF) were found in our present study with indescribable. However, all of the positive samples for *O. viverrini*-like egg were confirmed by PCR. Hence, the proportion of MIF and *O. viverrini* in this area and the involved factors need to be studied further.

For the other intestinal parasites, *Taenia* spp. was the second-highest prevalence. The human can get an infection of this parasite by eating raw pork or beef containing infective cysticercus and mostly develops intestinal taeniasis [45]. Our finding prevalence (0.42%) was lower than previous reports of *Taenia* spp. in Thailand. The nationwide survey in 2014 revealed 0.7% national prevalence with 1.6% in the northeast [16]. However, the prevalence can be varied when spotting in different regions [18, 39, 46]. The lowering prevalence of this parasite in this area suggested that the risky behavior may not be famous or will be altered in the population habits in this region. Another factor contributing to lowering the prevalence will be the vegetation system in Thailand has also been changed from manure to chemical fertilizer.

For the STHs, *T. trichiura* and hookworms, the prevalence were only 0.1 and 0.2%, respectively, that is a very low incidence of STHs in this region. The prevalence of STHs in Thailand is continuously declined even in the southern area due to sanitary improvement and also the educational campaigns for parasitic prevention all over the country. However, the prevalence can be a bit increased if specific or molecular techniques are used [6, 19, 22, 47, 48]. Our results suggested that the STHs will not be the problem of this area if the people are taking care of their sanitation, particularly, wearing shoes when going outside and eating of well-cooked foods.

For the epidemiological findings, our present study found that gender is insignificantly related to parasitic infection in this area. However, the males have a higher risk than females (6.39 and 5.47%, respectively) which corresponded to several previous studies [19, 38, 49]. However, this finding suggested that gender is not only the factor contributing high risk of parasitic infection, especially in the endemic areas where the consumption behavior between male and female are not different. Another insignificant factor from our study was the age of volunteering participants, our result also suggested that age is not a significant factor contributing to the parasitic infection. It is in contrast to many studies that mostly established that the elderly has a higher risk than the other ages due to immunity declining [18–20, 50–52]. On the contrary, our result stipulated that the age between 21–40-year-old has the highest risk of infection but the elderly has the lowest risk. It is possible due to the age of 21–40-year-old is an active age that outgoing for working in the rice field, fishing in the lake that has high possibility to do risky behaviors whereas elderly is mostly stayed at home and has a very low chance to eating of raw fish, pork, or beef. It might be the reason why the active age was greater prevalence than the other ages. The limitation of our study was missing the sociodemographic data, especially behaviors, however, the geographic finding was strongly evidenced enough to indicate the risk of people in this area.

## Conclusion

In conclusion, our present study indicated that *O. viverrini* infection still remains a major health problem in Thailand, especially in the northeastern region, although the prevalence has continuously declined. Our study demonstrated the crucial outcome that the house locations nearby the water reservoirs such as lakes, rivulets, canals, and rivers, provide high feasibility of *O. viverrini* infection other than gender and age. This finding could bring to the prospective strategy, especially sanitation improvement and education to limit the infections and the chance of cholangiocarcinoma in the future.

## Data Availability

The datasets generated and analyzed during the current study are not publicly available due to privacy and ethical restrictions but are available from the corresponding author upon reasonable request.

## Abbreviations

OV: *Opisthorchis viverrini*
TN: *Taenia* Spp.
HW: Hookworm
TT: *Trichuris trichiura*
ES: *Echinostoma* Spp.
PECT: PBS-ethyl acetate concentration technique
